# A comparison of the efficacy of darifenacin alone vs. darifenacin plus a Behavioural Modification Programme upon the symptoms of overactive bladder

**DOI:** 10.1111/j.1742-1241.2008.01714.x

**Published:** 2008-04-01

**Authors:** M B Chancellor, F Kianifard, E Beamer, L Mongay, U Ebinger, G Hicks, A DelConte

**Affiliations:** 1 Department of Urology, University of Pittsburgh School of Medicine Pittsburgh, PA, USA; 2 Novartis Pharmaceuticals Corporation East Hanover, NJ, USA; 3 Genaera Corporation, Plymouth Meeting Philadelphia, PA, USA

## Abstract

**Purpose:**

This study assessed the benefit of adding behavioural modification to darifenacin treatment for overactive bladder (OAB).

**Materials and methods:**

The ABLE trial was a randomised, open-label, parallel-group, multicentre study of 12 weeks of darifenacin treatment [with voluntary up-titration from 7.5 mg once daily (qd) to 15 mg qd at week 2] alone or in combination with a Behavioural Modification Programme (BMP) for men and women with dry or wet OAB. Efficacy was assessed as the change in the number (per day) of micturitions (primary variable), urge urinary incontinence (UUI) episodes, urgency episodes, pads used and nocturnal voids. Health-related quality of life (HRQoL) was also evaluated. Tolerability and safety assessments included adverse events and the number of discontinuations.

**Results:**

Of 592 patients screened, 395 were randomised, 190 to darifenacin alone and 205 to darifenacin + BMP. At baseline, the majority of subjects were dry (mean 2.8 and three UUI episodes per day in the darifenacin and darifenacin + BMP groups respectively). At study end, darifenacin alone and darifenacin + BMP both produced significant reductions from baseline in median numbers of micturitions, UUI episodes, urgency episodes and nocturnal voids (all p < 0.05), but not in the number of pads used. HRQoL also improved. There were no significant differences between treatment groups in efficacy or HRQoL variables.

**Conclusions:**

Darifenacin treatment provides a degree of normalisation of micturition variables and improvement in HRQoL that cannot be further enhanced by behavioural therapy of the type used in this study. Whether behavioural modification would add benefit over darifenacin treatment in patients with more pronounced incontinence problems remains to be determined.

**What's known**

- Behavioural therapy is usually the first step in managing overactive bladder, followed by pharmacological therapy with antimuscarinic drugs that inhibit detrusor activity.
- Both therapies are known to be effective independent treatments for OAB.
- Few trials have assessed the value of combining behavioural and pharmacological therapy.

**What's new**

- The study findings support the use of darifenacin as an effective agent for reducing the symptoms of OAB and improving health-related quality of life.
- The data do not support the hypothesis that behavioural modification of the type used in this study, adds any significant benefit over that achieved with darifenacin alone for treatment of the clinical symptoms of OAB, at least in our study population.

## Introduction

Overactive bladder (OAB) is characterised by urgency with or without urge incontinence, usually with urinary frequency and nocturia (1). OAB is a common condition that affects millions of people worldwide and OAB-associated urge urinary incontinence (UUI, ‘wet’ OAB) is especially prevalent in older women (2). This chronic condition significantly impairs quality of life and can seriously degrade various aspects of daily living (3).

Behavioural therapy is usually the first step in managing OAB, followed by pharmacological therapy with antimuscarinic drugs that inhibit detrusor activity. Behavioural programmes typically utilise bladder training, pelvic floor muscle training or a combination of the two techniques to reduce the number of incontinence episodes, controlling sensations of urgency and changing voiding habits. Behavioural training has been shown to have benefit for reducing UUI in a number of clinical studies (4–8) and offers an alternative to drugs and surgery for the treatment of OAB (9).

Although the exact means by which behavioural training reduces UUI episodes is unclear, the physiological mechanisms involved in producing the beneficial effects are likely to be different from those of antimuscarinic drugs (9). This observation suggests that the combination of behavioural training and antimuscarinic treatment may have additive benefit for the management of OAB.

This study was designed to assess the efficacy, safety and tolerability of darifenacin, an antimuscarinic agent with a high relative affinity for the M_3_ muscarinic receptor (10), alone or in combination with a Behavioural Modification Programme (BMP) for treating symptoms of OAB. In an attempt to reflect real clinical practice, the study included both dry and wet patients.

## Patients and methods

This study was conducted in compliance with the human experimentation guidelines of the US Department of Health and Human Services and the Helsinki Declaration, 1975, last amended in Edinburgh, Scotland, 2000. Written informed consent was obtained from each study participant.

### Patient population and demographics

This was a randomised, open-label, parallel-group, multicentre study, conducted between 23 May 2005 and 8 February 2006. Male and female patients ≥ 18 years old with symptoms of OAB for at least 6 months were enrolled in 64 US centres. Inclusion criteria for this study were ≥ 8 micturitions on average per day, ≥ 2 episodes of UUI on average per day and/or ≥ 2 episodes of urgency on average per day. Exclusion criteria included use of any drug that could affect bladder function within 2 weeks prior and during the study, participation in any formal bladder-training programme within 30 days of screening, predominant stress urinary incontinence, and any bladder or neurological condition that could affect urinary bladder function or in which use of anticholinergic drugs was contraindicated.

### Study design

**Figure 1:**
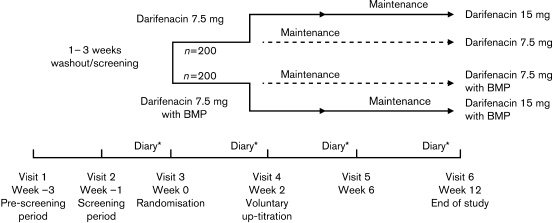
A schematic of the study design. BMP, Behavioural Modification Programme. *3-day paper diary completed 1 week prior to clinic visit

This multicentre US study was designed to evaluate the efficacy, safety and tolerability of darifenacin administered alone or in combination with BMP. The study consisted of a 2-week washout and 1-week screening period followed by 12 weeks of treatment (Figure 1). Patients were instructed to record symptoms of OAB in a bladder diary for the three consecutive days immediately preceding each evaluation (at randomisation, and at study weeks 2, 6 and 12), and patients meeting inclusion and exclusion criteria were randomly assigned to receive darifenacin treatment either alone or in conjunction with a BMP. Randomisation was performed by the sponsor using a validated, computer-generated randomisation scheme. The randomisation scheme was reviewed by the sponsor's Quality Assurance group and locked after approval.

Darifenacin was taken orally once daily (qd) in the morning with an adequate amount of fluid. Patients assigned to the darifenacin plus BMP group received brochures on modification of diet and daily habits. These patients were trained in pelvic muscle exercises and urgency control techniques using materials (listed in Table 1) developed in conjunction with the National Association for Continence. The BMP comprised a simple regimen for which training could be given in a primary physician's office, including timed voiding, dietary modifications and Kegel-type exercises, and was meant to reflect real-life clinical practice. Complex regimens that would require extensive training or a trained physical therapist were purposefully avoided because that would not be practical in primary care. Study staff at each site reviewed each component of the BMP with patients.

**Table 1:** Components of the Behavioural Modification Programme

**Pamphlets**
Take control-incontinence: diagnosis, treatment and management
Diet and daily habits: can this affect your bladder or bowel control?
Pelvic muscle exercises: exercises specifically for the pelvic floor
Additional behavioural strategies for success: urge control
**Additional materials**
Motivational video
Exercise audio CD
Pelvic floor training manual

All materials © NAFC, 2005 (http://www.nafc.org).

Patients in both treatment groups initially received darifenacin 7.5 mg qd, and were allowed to increase their darifenacin dose to 15 mg qd after 2 weeks of treatment if the patient required greater efficacy and the current dose was well tolerated, or remained on 7.5 mg qd throughout the study.

### Efficacy, safety and tolerability assessments

The primary efficacy variable was the change from baseline in average number of micturitions per day. Week 12 was the primary analysis time point, with additional analyses conducted for weeks 2 and 6. Secondary efficacy variables included changes from baseline per day in the following: average number of UUI episodes; average number of urgency episodes; average number of urinary incontinence pads used; average number of nocturnal voids because of OAB.

In addition, health-related quality of life (HRQoL) was evaluated by the Overactive Bladder Questionnaire (OAB-q) at baseline, Weeks 6 and 12 and patient satisfaction with treatment at week 12 was evaluated using the Overactive Bladder Satisfaction with Treatment Questionnaire (OAB-SAT-q). Results for the psychometric properties of the OAB-SAT-q questionnaire and analysis of the modified Female Sexual Function Index will be reported elsewhere. Safety and tolerability were assessed by recording adverse events (AEs) by severity and relationship to study drug, and discontinuations.

### Statistical methods

Statistical analyses were performed using SAS, Version 8.2 (Cary, NJ, USA). All statistical tests employed a two-sided significance level of 0.05. All statistical analyses were performed on the intent-to-treat (ITT) population, which includes all randomised patients who took at least one dose of study medication. For those patients who discontinued from the study or whose diary entries at week 12 were missing, the ITT analysis was performed using the last-observation-carried-forward method. If the diary entries for any variable were missing for at least two of the required 3 days at weeks 6 and 12, the average from the previous visit was carried forward and used in the summaries and analyses. If all diary entries for a particular variable were missing during baseline or week 2, the corresponding average value was considered missing.

All efficacy variables were analysed using the stratified Wilcoxon rank-sum test (van Elteren's test), adjusted for baseline severity (three categories defined by the baseline 33rd and 67th percentiles). The difference between the two treatment groups was expressed as the Hodges–Lehmann point estimate and the associated 95% confidence interval (CI) (11). Planned subgroup analyses of the efficacy variables were performed for patients who were ≥ 65 years old.

Changes from baseline in the total score and each dimension/domain score of OAB-q and OAB-SAT-q were analysed using an analysis of covariance model with treatment group, age, gender and baseline (if applicable) as explanatory variables.

For the primary efficacy objective, it was assumed that the probability of observing the same or a more favourable response on darifenacin + BMP relative to darifenacin alone, measured using the primary efficacy variable, was in the range of 0.58–0.59. With 200 patients per group, the power to detect a difference between the two groups was 79–87%, depending on the assumed probability and using a two-sided Wilcoxon rank-sum test with a significance level of 0.05 (12).

## Results

### Patient disposition

**Figure 2:**
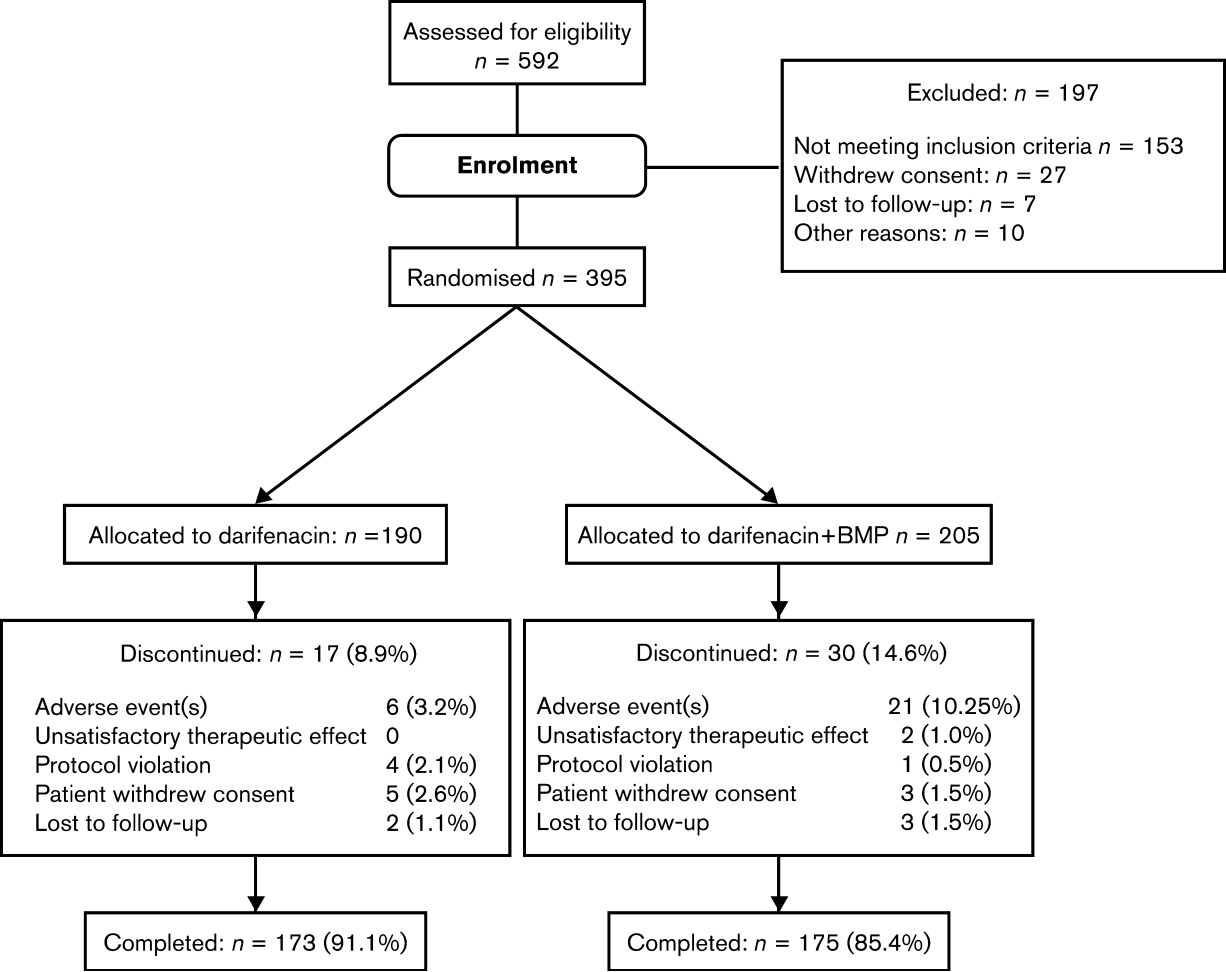
Patient disposition and flow

Of 592 patients screened, a total of 395 patients were randomised, 190 to darifenacin alone, and 205 to darifenacin + BMP. There were 47 discontinuations (17 darifenacin, 30 darifenacin + BMP; Figure 2), most commonly because of AEs (3.2% darifenacin alone, 10.2% darifenacin + BMP). Five patients (1.3%; four darifenacin alone, one darifenacin + BMP) were discontinued from the study because of protocol violations: non-compliance with study drug (2), incorrect procedure/procedure not done (2), not meeting the inclusion/exclusion criteria (1).

### Patient demographics and baseline values

The two treatment groups were comparable (Table 2), with an average age of 57–59 years and predominantly female (89%). The mean age of the subgroup of patients ≥ 65 years old (*n* = 134, 34%) was 72.9 years and this group was also predominantly female (84%).

**Table 2:** Patient demographics

	Darifenacin	Darifenacin + BMP
Randomised (*n*)	190	205
Mean (SD) age	57.4 (13.06)	58.5 (14.64)
Female (%)	90	88.3
**Race (%)**		
White	90	88.3
Others	10	11.7

BMP, Behavioural Modification Programme; SD, standard deviation.

Baseline values of the primary and secondary efficacy variables are summarised in Table 3. To assess the severity of incontinence at baseline, the distribution of average number of UUI episodes per day at baseline was also examined in both the overall and ≥ 65-year-old subgroup (Table 4). At baseline, the majority of subjects in both treatment groups and age subgroups were dry or had few UUI episodes (> 0 to < 7 episodes).

**Table 3:** Baseline values of efficacy variables

	Darifenacin	Darifenacin + BMP
		
	*n* = 189	*n* = 204
**Micturitions per day**		
Mean ± SD	11.92 ± 3.03	11.75 ± 3.37
Median	11.33	11.33
**UUI episodes per day**		
Mean ± SD	2.78 ± 2.57	3.00 ± 2.56
Median	2.33	2.58
**Urgency episodes per day**		
Mean ± SD	10.88 ± 3.80	10.58 ± 4.00
Median	10.67	10.33
**Pads used per day**		
Mean ± SD	1.12 ± 1.93	0.99 ± 1.67
Median	0	0
**Nocturnal voids per day**		
Mean ± SD	1.77 ± 1.43	1.87 ± 1.35
Median	1.67	1.67

BMP, Behavioural Modification Programme; UUI, urge urinary incontinence; SD, standard deviation.

**Table 4:** Baseline distribution of average number of UUI episodes per day in the total intent-to-treat population and ≥ 65-year-old subgroup

	Darifenacin	Darifenacin + BMP
**Average number of UUI episodes per day at baseline, *n* (%)**		
Total population, *n*	189	204
0 episodes	36 (19.1)	29 (14.2)
> 0 to < 7 episodes	138 (73.0)	155 (76.0)
≥ 7 to < 14 episodes	15 (7.9)	20 (9.8)
≥ 14 episodes	0 (0.0)	0 (0.0)
≥ 65 years old	53	81
0 episodes	9 (17.0)	8 (9.9)
> 0 to < 7 episodes	40 (75.5)	63 (77.8)
≥ 7 to < 14 episodes	4 (7.5)	10 (12.3)
≥ 14 episodes	0 (0.0)	0 (0.0)

BMP, Behavioural Modification Programme; UUI, urge urinary incontinence.

### Efficacy

**Figure 3:**
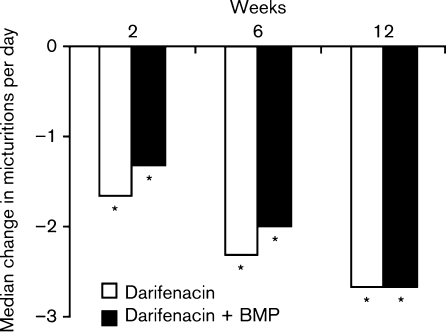
Median change from baseline in average number of micturitions per day over the 12 weeks of treatment with darifenacin or darifenacin plus Behavioural Modification Programme (BMP) for the intent-to-treat (ITT) population. There was no significant difference between the responses to the two treatments at any of the assessment time points. *p < 0.05 vs. baseline

The median of the average number of micturitions per day in the ITT population decreased from baseline (11.33 in both treatment groups) to 8.67 for darifenacin alone and 8.67 for darifenacin + BMP at week 12. Thus, the median change from baseline to week 12 in the average number of micturitions per day was −2.7 (95% CI: −3.3, −2.0) for darifenacin alone and −2.7 (95% CI: −3.0, −2.3) for darifenacin + BMP. Significant reductions were recorded at each time point (Figure 3), but did not differ significantly between treatment groups. Thus, near-normalisation of micturition frequency was achieved at week 2 (median 9.67 micturitions per day in both groups), with only small further improvements at weeks 6 or 12 (median 8.67 micturitions per day in both groups at both time points). Results were similar for the ≥ 65-year-old subgroup; the average number of micturitions per day decreased in both darifenacin (median change to week 12: −2.7; 95% CI: −3.3, −1.7) and darifenacin + BMP (−2.8; 95% CI: −3.3, −2.0) groups with no statistically significant difference between the groups.

At week 12, treatment with darifenacin or darifenacin + BMP both resulted in significant reduction from baseline in the median number of UUI episodes, urgency episodes and nocturnal voids for the ITT population (i.e. including both wet and dry patients), but the change in the number of pads used vs. baseline was not significant (Table 5). The reductions in all parameters were not significantly different between the two treatments.

**Table 5:** Summary of results for primary and secondary efficacy variables after 12 weeks of treatment with darifenacin or darifenacin + BMP for the ITT population

	Darifenacin	Darifenacin + BMP	
			
	*n* = 184	*n* = 195	Comparison
**ΔMicturition episodes per day**			
Mean ± SD	−2.96 ± 2.91	−2.82 ± 2.87	
Median (95% CI)	−2.67 (−3.33, −2.00)	−2.67 (−3.00, −2.33)	
H–L difference (95% CI)			0.00 (−0.67, 0.33)
p-value			0.681
**ΔUUI episodes per day**			
Mean ± SD	−1.89 ± 2.29	−2.10 ± 2.32	
Median (95% CI)	−1.33 (–2.00, −1.00)	−2.00 (−2.00, −1.33)	
H–L difference (95% CI)			0.33 (−0.00, 0.67)
p-value			0.268
**ΔUrgency episodes per day**			
Mean ± SD	−2.87 ± 3.59	−2.68 ± 3.54	
Median (95% CI)	−2.33 (−3.00, −1.67)	−2.67 (−3.00, −2.00)	
H–L difference (95% CI)			0.00 (−0.67, 0.67)
p-value			0.882
**ΔPads used per day**			
Mean ± SD	−0.72 ± 1.54	−0.61 ± 1.28	
Median (95% CI)	0 (0, 0)	0 (0, 0)	
H–L difference (95% CI)			0.00 (0.00, 0.00)
p-value			0.978
**ΔNocturnal voids per day**			
Mean ± SD	−0.65 ± 1.26	−0.67 ± 1.21	
Median (95% CI)	−0.67 (−0.67, −0.33)	−0.67 (−0.67, −0.33)	
H–L difference (95% CI)			0.0 (−0.00, 0.33)
p-value			0.315

BMP, Behavioural Modification Programme; ITT, intent-to-treat; Δ, change from baseline; UUI, urge urinary incontinence; H–L, Hodges–Lehmann. p-values are based on Wilcoxon rank-sum test, adjusting for baseline severity.

### Health-related quality of life

Both the darifenacin alone and darifenacin + BMP groups showed improvement in total OAB-q and total OAB-SAT-q scores at week 12 indicating improved HRQoL with both treatments. The differences between the two groups for both the OAB-q and OAB-SAT-q at week 12 were not significant.

### Safety and tolerability

Adverse events were comparable between treatments. The most frequently reported AEs were constipation (18.5%), dry mouth (25%), urinary tract infection (4.8%), and headache (3.8%) and most were mild to moderate in intensity. A substantial proportion (53.8%) of AEs was considered to be unrelated to treatment. In total, 27 patients (6.8%) discontinued the study because of an AE, eight patients (2%) because of constipation and seven patients (1.8%) because of dry mouth.

## Discussion

This study was designed to collect data on efficacy, safety and tolerability of darifenacin administered alone or in conjunction with BMP. As the addition of BMP to darifenacin did not provide any significant additional improvement on OAB symptoms, these data suggest that darifenacin alone may provide a degree of improvement that cannot be further enhanced by behavioural therapy. Although both behavioural and drug therapies are known to be effective treatments for OAB independently, there are relatively few trials assessing the value of combining these two modes of treatment. Whether behavioural therapy might have an additive benefit with drug treatment remains to be confirmed, with inconsistent findings reported to date (13–16).

Two studies reported no increase with combined anticholinergic and bladder training, compared with bladder retraining without anticholinergic therapy (13,14). In contrast, two studies found that the combination of drugs and behavioural training can produce more benefit than either therapy alone (15,16). In one study, community-dwelling women with urodynamic evidence of bladder dysfunction, were randomised to behavioural treatment, oxybutynin or placebo and offered the opportunity to crossover into combined therapy if not satisfied after 8 weeks (15). Twenty-seven subjects (41.5%) from drug therapy alone and eight subjects from behavioural treatment alone crossed to combined treatment, with both groups achieving an additional improvement in the reduction of incontinence. However, these results cannot be considered definitive based on the small sample size. The second (multicentre, single-blind) study compared tolterodine treatment with and without the addition of written information about bladder training (16). In this study, the effect on voiding frequency and volume voided was enhanced by the combination, but there was no further improvement in urgency or incontinence episodes. Recently, a Cochrane review evaluated randomised or quasi-randomised controlled trials of treatment with anticholinergic drugs for OAB syndrome or UUI in adults, in which at least one management treatment group involved a non-drug new therapy, including BMPs (17). The results regarding bladder training favoured a combination of anticholinergics with bladder training compared with anticholinergics during treatment even if the difference was not statistically significant.

In the present study, the addition of BMP to darifenacin produced no greater reduction in micturition frequency or UUI than was seen with darifenacin alone. One possible explanation for this is that the mechanisms underlying the two treatment effects, although different, may have a common single outcome of decreasing detrusor contraction, or increasing detrusor stability. Comparing the specific nature of the selective M_3_ receptor blockade provided by darifenacin, with the more general behavioural (both physiological and psychological) approach with BMP, this would seem unlikely. Indeed, both treatments are likely to do more than simply reduce detrusor activity. For example, it has been suggested that additional direct effects of antimuscarinic agents play a role in their overall therapeutic effects, and that the reduction of sensory symptoms such as urgency, may be mostly independent of their effect upon voiding contractions (18–20).

Another possibility for the lack of additional benefit of BMP was inadequate compliance with the BMP, although this was not formally assessed in our study. In a previous retrospective study of 123 women with UUI, 55% of the women either never started the bladder retraining programme or discontinued it before study completion illustrating the difficulties with compliance faced by clinical trials (20).

Interestingly, Burgio et al. (15) performed a study in older women (mean age = 69.3 years) and demonstrated some benefit of combining drug and behavioural therapy. The patient population was entirely female, older than in the present study and employed biofeedback techniques as part of the training regimen (15). In comparison, the present study population was mostly female (approximately 90%), and the analysis of older patients (≥ 65 years) did not yield any significant findings to suggest an additional benefit of BMP in older patients, although of note, the study was not powered to detect an effect in the older patient subgroup.

An additional consideration may be that the effectiveness of BMP may differ between different types of BMP. For example, the completion of patient diaries may themselves confer (albeit to a small extent) a degree of behavioural training. In this study, such an effect would have occurred for both patient groups, thereby reducing the potential for further benefit by addition of BMP. For formal BMP programmes, effectiveness would be expected to increase with intensity of training (with personal tuition expected to yield better results than paper/video instructions alone), and with subsequent monitoring and reinforcement of training by the caregiver. In the present study, training occurred only at baseline and week 2, and there are no data to confirm consistent application of the behavioural recommendations. This approach is expected to be consistent with that used in ‘real-life’ clinical settings.

Finally, it may be that behavioural modification training is more successful in patients with more pronounced incontinence problems. In our trial population the baseline mean number of UUI episodes per day was 2.8 in the darifenacin group, and 3.0 in the darifenacin + BMP group. Thus, there was limited scope for improvement in either group, and this is also demonstrated by the finding that near-normalisation of micturition frequency was already achieved by week 2. This relatively ‘dry’ profile of patients may therefore have further contributed to the non-significance of adding BMP to darifenacin treatment in this study.

In summary, the findings of the present study support the use of darifenacin as an effective agent for reducing the symptoms of OAB and improving HRQoL. However, the data do not support the hypothesis that behavioural modification of the type used in this study, adds any significant benefit over that achieved with darifenacin alone for treatment of the clinical symptoms of OAB, at least in our study population. Further studies are necessary to define the combination of patient populations, drugs and behavioural modification regimen that provide optimal reduction of OAB symptoms.

## References

[1] Abrams P, Cardozo L, Fall M (2002). The standardization of terminology of lower urinary tract function: report for the standardization sub-committee of the International Continence Society. Neurourol Urodyn.

[2] Rovner ES, Wein AJ (2002). Incidence and prevalence of overactive bladder. Curr Urol Rep.

[3] Abrams P, Kelleher C, Kerr LA, Rogers RG (2000). Overactive bladder significantly affects quality of life. Am J Manag Care.

[4] Burgio KL, Whitehead WE, Engel BT (1985). Urinary continence in the elderly: bladder-sphincter biofeedback and toileting skills training. Ann Intern Med.

[5] Burton JR, Pearce KL, Burgio KL, Engel BT, Whitehead WE (1998). Behavioral training for urinary incontinence in elderly ambulatory patients. J Am Geriatr Soc.

[6] Elser DM, Wyman JF, McClish DK, Robinson D, Fantl JA, Bump RC (1999). The effect of bladder training, pelvic floor muscle training, or combination training on urodynamic parameters in women with urinary incontinence. Neurourol Urodyn.

[7] Burgio K L, Goode PS, Locher JL (2002). Behavioral training with and without biofeedback in the treatment of urge incontinence in older women. JAMA.

[8] Goode PS (2004). Behavioral and drug therapy for urinary incontinence. Urology.

[9] Burgio KL (2002). Influence of behavior modification on overactive bladder. Urology.

[10] Napier C, Gupta P (2002). Darifenacin is selective for the human recombinant M_3_ receptor subtype. Neurol Urodyn.

[11] Desu MM, Raghavarao D (2003). Nonparametric Statistical Methods for Complete and Censored Data.

[12] Noether G (1987). Sample size determination for some common nonparametric tests. J Am Stat Assoc.

[13] Fantl JA, Hunt WG, Dunn LJ (1981). Detrusor instability syndrome: the use of bladder retraining with and without anticholinergics. Am J Obstet Gynecol.

[14] Ouslander JG, Schnelle JF, Uman G (1995). Does oxybutynin add to the effectiveness of prompted voiding for urinary incontinence among nursing home residents? A placebo-controlled trial. J Am Geriatr Soc.

[15] Burgio KL, Locher JL, Goode PS (2000). Combined behavioral and drug therapy of urge incontinence in older women. J Am Geriatr Soc.

[16] Mattiasson A, Blaakaer J, Hoye K, Wein AJ (2003). Simplified bladder training augments the effectiveness of tolterodine in patients with an overactive bladder. BJU Int.

[17] Alhasso AA, McKinlay J, Patrick K, Stewart L (2006). Anticholinergic drugs versus non-drug active therapies for overactive bladder syndrome in adults. Cochrane Database Syst Rev.

[18] Andersson KE, Wein AJ (2004). Pharmacology of the lower urinary tract: basis for current and future treatments of urinary incontinence. Pharmacol Rev.

[19] Andersson KE, Yoshida M (2003). Antimuscarinics and the over active detrusor – which is the main mechanism of action?. Eur Urol.

[20] Finney SM, Andersson K-E, Gillespie JI, Stewart LH (2006). Antimuscarinic drugs in detrusor overactivity and the overactive bladder syndrome: motor or sensory actions?. BJU Int.

